# Large body size constrains dispersal assembly of communities even across short distances

**DOI:** 10.1038/s41598-018-29042-0

**Published:** 2018-07-19

**Authors:** Richard I. Bailey, Freerk Molleman, Chloe Vasseur, Steffen Woas, Andreas Prinzing

**Affiliations:** 1 Université de Rennes 1/CNRS; Ecosystèmes, Biodiversité, Evolution (ECOBIO); Campus de Beaulieu, 35042 Rennes, France; 2 Institute of Ecology and Earth Sciences, University of Tartu, Vanemuise 46, EE-51014 Tartu, Estonia; 3 Vanasiri Evolutionary Ecology Lab, IISER-TVM Centre for Research and Education in Ecology and Evolution (ICREEE), Indian Institute of Science Education and Research, Thiruvananthapuram, Kerala India; 4 Department of Systematic Zoology, Institute of Environmental Biology, Faculty of Biology A. Mickiewicz University, Umultowska st 89, PL-61-614, Poznań, Poland; 5 Staatliches Museum für Naturkunde Karlsruhe, Abteilung Zoologie, Postfach 111364, 76063 Karlsruhe, Germany

## Abstract

Dispersal limitation has been considered to decrease with body size in animals and to be an important factor limiting community assembly on spatially isolated patches. Here we hypothesize that for flightless bark-dwelling oribatid mites dispersal limitation onto young trees might increase with body size (due to a decrease in aerial dispersal capacities), and it might occur even within a spatially contiguous forest canopy. We suppressed dispersal limitation towards branches from young trees by physically connecting them to branches from old trees and analyzed the impacts on community composition, accounting for branch microhabitat variables. Suppression of dispersal limitation increased community evenness and mean body size of mites on branches from young trees. Across all species, large species body-size corresponds to an abundance increase after suppression of dispersal limitation. Consistently, on no-contact control branches, mite body-sizes were larger on branches from old compared to young trees. Our study suggests that colonization/performance trade-offs might affect community assembly even across seemingly contiguous habitats. Overall, a previously underappreciated factor selecting against large body size in flightless canopy-dwelling invertebrates might be that large bodies makes these invertebrates fall faster and disperse less, not more.

## Introduction

Islands are places that are hard to reach and dispersal may hence limit the assembly of species communities on islands (we define community assembly as the processes controlling which species establish at which abundances in a community). Island biogeography usually focuses on oceanic islands or habitat islands of hectares in size and isolated by kilometres^1^. However, dispersal limitation –the limitation in the ability of organisms to reach a given locality - might affect community assembly also at much smaller scales: Large organisms may be hosts for small, flightless organisms that live inside or on the host and are unable to migrate actively between hosts, even if these hosts are spatially proximate^2,3^. Possible examples include macroparasites living inside social mammals^4^ or mites living on the bark of forest trees^5^. For such communities, individual hosts might act as islands, limiting colonization even across the short distances among hosts. Effects of such dispersal limitation on communities on hosts are particularly likely if hosts are young, because time for colonization was short, and because young hosts are small, harboring particularly small colonizer populations facing particularly high risks of extinction that would need to be compensated by particularly frequent recolonizations^3^.

Dispersal assembly of local communities on islands is often described by “neutral” models that treat dispersal as a random process in which differences between species are unimportant for the establishment success of species^6,7^. However, dispersal limitation is to some extent deterministic as species differ in their capacity to disperse, and this capacity may be negatively traded off against performance such as competitiveness or stress tolerance^8–11^. Specifically, for macroscopic animals, dispersal limitation is often considered to increase with a decrease in body size as smaller animals spend proportionally more energy for locomotion and have proportionally less energy at their disposal^12–14^. However, in the case of flightless colonizers on hosts such as in many mite species, dispersal is often passive by floating through the air among hosts. Dispersal is hence not limited by available energy, but by gravity. Smaller organisms float greater distances than larger ones^15,16^ resulting in a possible *in*crease of dispersal limitation with size. However, these studies showed that larger mites fall faster, but did not address any effects on community assembly or species establishment. For wind-dispersed plant seeds, smaller seeds are known to be more likely to reach distant patches^17^ and references herein, while common wisdom suggests that among macroscopic animals larger individuals disperse farther^12–14^. Overall, dispersal limitation will depend on body size, but the direction of this body-size dependency remains unclear for animals colonizing hosts.

There are three major and valuable approaches that have been applied to test for dispersal limitation, but all suffer from certain shortcomings. First, dispersal limitation has often been inferred from decreasing community similarity with increasing spatial distance^18^. However, also the similarity in habitat niches may decline with distance. Observational testing of niche vs. dispersal-based community assembly hence relies on a comprehensive quantification of all pertinent niche parameters, including small-scale microhabitat conditions, which is bound to be very difficult^19^. Second, dispersal limitation has been tested experimentally by transferring individuals, in particular adding seeds^20,21^. This approach is powerful (at least in plants) as it avoids confounding dispersal with habitat properties. However, if adding seeds increases species richness this might not only reflect dispersal limitation of seeds among distant habitat patches - it might also reflect limited local recruitment through reproduction, e.g. due to insufficient resources for seed production. This shortcoming may be critical because dispersal- and recruitment limitation have different consequences^22,23^. Third, dispersal limitation has been tested experimentally by cutting a contiguous habitat into disconnected habitat patches^24^, but this helps little in deciphering the importance of dispersal limitation between naturally disconnected habitat patches where species may have evolved solutions to successfully disperse. A fourth, and perhaps most straightforward approach, would be to directly suppress dispersal limitation, notably in young, non-equilibrium habitat islands. This would require connecting formerly disconnected habitat islands so that frequent foraging movements rather than rare dispersal events permit to move between the habitat islands. Organisms would not be forced to move into isolated patches, but could choose on their own. To our knowledge, this has not been done so far. Notably, this is different from artificially disconnecting naturally contiguous patches and then experimentally creating corridors between the experimentally created fragments^24^.

One case of spatially adjacent “islands” on which neutral or body-size dependent dispersal limitation might be important are tree crowns in contiguous forest canopy and their bark-living oribatid mite communities^25^. These mite species live on the bark surface and use cryptogams such as lichens and algae for both food and shelter. Most of these mite species can disperse among trees only by passively floating through the air^26–28^ as the ground is not suitable habitat for most tree crown-dwelling species^25,28–30^. Tree-to-tree dispersal has not been studied previously but is consistent with observed patterns of beta diversity^31^. Scarcity of immigration can only slowly be compensated by high *in situ* reproduction as these mites have slow intrinsic growth rates, with life spans of often more than one year^32^. Taken together, dispersal among tree crowns may be a limiting factor in the assembly of bark mite communities, in particular on tree crowns that are relatively young. Furthermore, younger trees tend to be smaller and mites on smaller trees may be more prone to local extinction, and thus younger trees may require re-colonization more often. The capacity of oribatid mites to float large distances decreases with increasing body size^15,16^ (for Phytoseiid Acari covering a similar range of body sizes) so that dispersal limitation should increase with body size. However, once large-bodied mites have succeeded in colonizing a tree crown, they might perform better than smaller mites as larger mites may more efficiently break up cortices of cryptogams^33^, and the small relative body surface reduces sensitivity to desiccation from unsaturated air in the canopy^34–36^.

We hypothesize that dispersal limitation in crown-dwelling oribatid mites operates even among the adjacent crowns of a mature forest canopy, and that this dispersal limitation increases rather than decreases with body size. We also hypothesize that performance of established mites increases with body size and populations of smaller mites would decline once larger, better performing mites colonize the tree crown.

**Figure 1:**
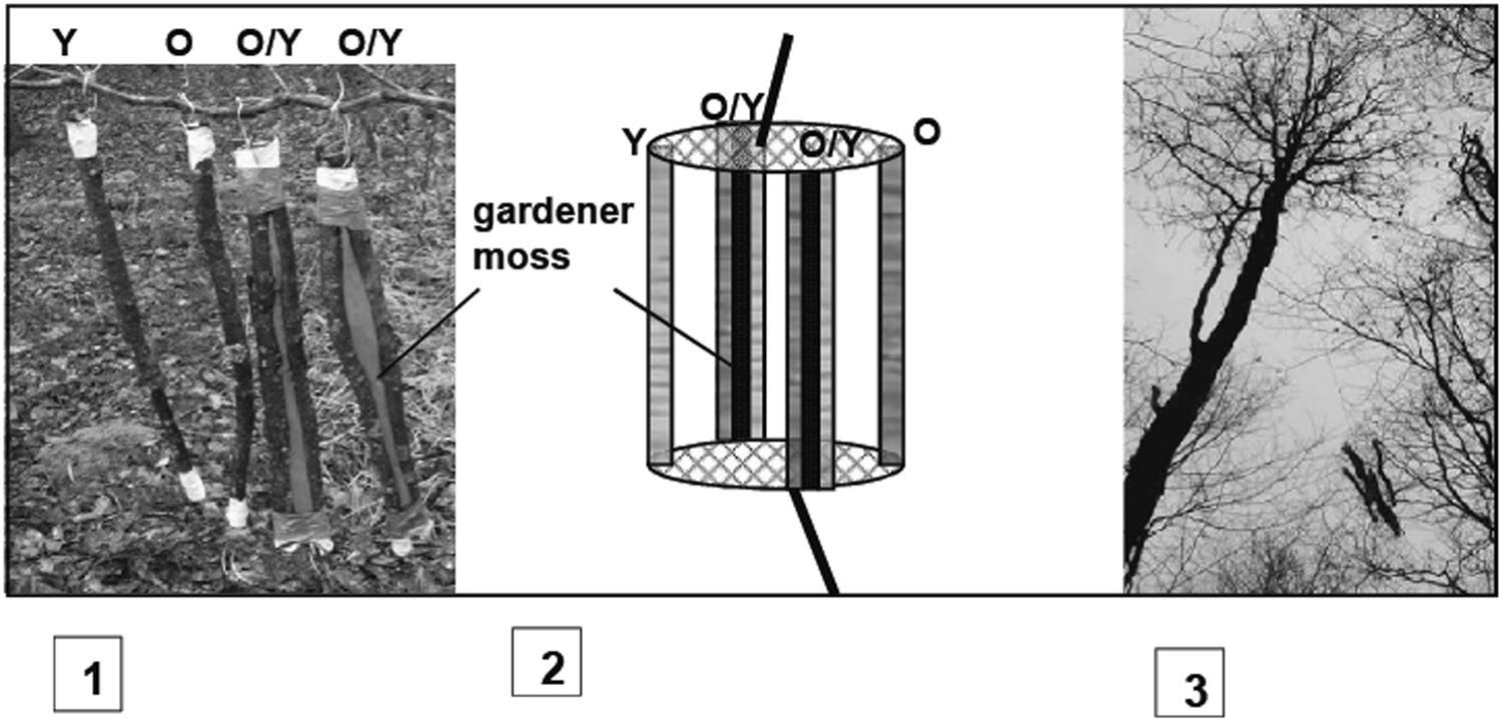
Experimental setup. 1: branches, with connection between branches ensured by floral foam, prior to installation in the canopy, 2: branches connected on wire support, 3: installation in the canopy. “O” and “Y” corresponds to branches from older and younger tree crowns.

To test the importance of dispersal limitation among directly adjacent habitat islands we suppressed dispersal limitation among old and young mature tree crowns of approximately 30 and >60 years. For this purpose we put branches taken from young crowns in contact with branches from old crowns (Methods; Fig. 1). We showed that, once branches were put in contact, mites could easily walk from branch to branch through short-term movements. We stress that only trees, and not branches, differed in age. If suppression of dispersal limitation resulted in redistribution of particular mite species towards young-crown branches, this indicated that prior to the treatment dispersal was limited and prevented such redistribution. Our approach minimized impact of variation in habitat properties. The experiment manipulated dispersal limitation while keeping habitat properties and microclimate constant. Moreover, sampling of branches minimized variation in habitat properties and ambient conditions to bark of peripheral branches of younger and older trees within the lower canopy of approx. 8 m height, of a mature, temperate forest. Finally, we tested for any remaining differences in microhabitat properties and accounted for microhabitat properties when testing for an effect of age. We recorded abundances per species, not just presence/absence, as abundances provide more fine-grained information and are affected by dispersal limitation^24,37,38^. We tested the predictions of our hypothesis: that suppression of dispersal limitation results in an increase of mean body size of oribatid mites and a decline of small-bodied species. Since the communities were dominated by two species of contrasting body size, we examined to what degree community-wide changes in mean body size reflect changes in abundances of these two most abundant species. We then explored the generality across all species, including rarer ones, testing whether species of larger body size become more abundant after suppression of dispersal limitation compared to small-bodied species. We also studied various community parameters that may change as a result of dispersal limitation^1,7^, notably species diversity, their total abundance, and evenness of abundances.

## Results

A total of 2363 individuals were found, including 514 juveniles, out of which only 8 could not be identified to species level. Table 1 lists abundances of species and their body size and arboral life style. Microhabitat composition (percent cover of cryptogams and bare bark) only slightly varied between young-tree versus old-tree branches, or between experimental treatments (Table 2) and differences were not significant (Table 3). Nevertheless, we below controlled for the influence of microhabitat by including all microhabitat variables when testing the influences of tree age and suppression of dispersal limitation on mite communities.

**Table 1:** Oribatid mite species found during this study, their mean body size and arboreal life style (0 = mainly ground living; 0.5 = both living at the ground [notably dead wood] and on bark/cryptogams; 0.75 = living in cryptogams or mainly arboreal; and 1 = arboreal) and the species’ mean abundances (per 60 cm branch) for each branch category (Y/O: young- vs old-crown branches; a/c: alone vs in contact with contrasting age class).

Species	Mean body size (µm)	Arboreal life style	Abundances			
			Ya (n = 8)	Yc (n = 16)	Oa (n = 8)	Oc (n = 16)
*Caleremaeus monilipes* (Michael, 1882)	424	0	0.250	0.313	0.000	0.063
*Camisia segnis* (Hermann, 1804)	865	0.75	2.375	0.625	1.875	1.375
*Carabodes labyrinthicus* (Michael, 1879)	505	0.5	0.500	1.313	0.375	0.250
*Cepheus pegazzaanoe* (Bernini & Nannelli, 1982)	700	0	0.000	0.000	0.125	0.000
*Cymbaeremaeus cymba* (Nicolet, 1855)	745	1	3.625	2.438	3.250	2.375
***Dometorina plantivaga*** **(Berlese, 1895)**	**442,5**	1	**13.750**	**16.938**	**16.375**	**9.063**
*Eupelops claviger* (Berlese, 1916)	700	0	2.000	2.625	5.625	3.438
*Liebstadia humerata* (Michael, 1888)	345	0.75	0.000	0.250	0.000	0.125
***Micreremus brevipes*** **(Michael, 1888)**	**290**	0.75	**35.125**	**27.250**	**11.375**	**17.500**
*Oppiella splendens* (C.L.Koch, 1841)	320	0	0.000	0.000	0.000	0.063
*Phauloppia lucorum* (C.L.Koch, 1841)	750	1	1.500	3.688	2.875	3.188
*Phauloppia pilosa* (Michael, 1888)	430	1	0.250	0.875	0.000	0.125
*Phthiracarus ferrugineus* (C.L.Koch, 1841)	722,5	0	0.000	0.063	0.000	0.000
*Phthiracarus starmineus* (C.L.Koch, 1841)	675	0.5	0.125	0.000	0.000	0.000
*Poroliodes farinosus* (C.L.Koch, 1840)	1050	0.5	0.000	2.375	0.000	0.000
*Ramusella elliptica* (Berlese, 1908)	245	0	0.000	0.000	0.125	0.000
*Xenillus discrepans* (Grandjean, 1936)	1007,5	0.5	0.000	0.000	0.125	0.000
*Zygoribatula exilis* (Nicolet, 1855)	380	1	0.000	0.063	0.000	0.000

In bold: dominant species (representing together 74% of total oribatid abundance). Note that for the rarer species, zero abundance will reflect rarity rather than complete absence from the tree.

**Table 2:** Percentage of cover (mean and range) of the different microhabitats types (cryptogams and bare bark) on young branches from young- and old-crown of mature oaks. N = 48.

	Young-crown branches				Old-crown branches			
	Alone		Connected		Alone		Connected	
	mean (%)	Range	mean (%)	range	mean (%)	range	mean (%)	range
	n = 8		n = 16		n = 8		n = 16	
Algae	64	(*28*–*95*)	54	(*20*–*93*)	60	(*27*–*94*)	51	(*20*–*93*)
Crustose lichens	19	(*5*–*39*)	21	*(4*–*51)*	17	(*6*–*42*)	19	(*3*–*56*)
Foliose lichens	2	(*0*–*6*)	2	(*0*–*5*)	2	(*0–8*)	2	(*0–15*)
Mosses	<1	(<*1*)	3	(*0–31*)	8	(*0*–*39*)	9	(*0*–*41*)
Bare bark	15	(*0*–*50*)	21	(*0*–*65*)	13	(*0*–*33*)	19	(*0*–*49*)

**Table 3:** Test of differences in microhabitat composition on young branches depending on crown age and the treatment of these branches (connected/alone). MANOVA, i.e. multiple dependent variables. Df factor/error = 5/24.

	Wilk’s lambda	F	p
Intercept	0.033	140.67	<0.0001
Young tree = 1	0.766	1.46	0.2381
Branch connected = 1	0.962	0.19	0.9631
Young tree = 1*Branch connected = 1	0.977	0.12	0.9878

### Dispersal limitation towards young tree-crowns decreased evenness

Neither richness nor abundance of oribatid mites depended on the age of the tree crown of origin, contact with branches from crowns of contrasting age, nor the interaction between the two (Table 4). Evenness, in contrast, was lower on branches from young crowns but increased after dispersal limitation was suppressed by putting them in contact with old-crown branches (Table 4).

**Table 4:** Characteristics of communities of oribatid mites on branches depending on: cryptogam cover (crustose lichens, foliose lichens, mosses, algae, bare bark); younger vs. older age of the crown of origin; the experimental suppression of dispersal limitation between these crowns by putting their branches into contact; and the interaction between age of the crown of origin and contact between branches.

Response	Variable	Parameter	Lower 95% CI	Upper 95% CI	P value
Total abundance	—	—			ns
Species richness	—	—			ns
Evenness (Pielou’s J)	Intercept	0.78	0.67	0.90	<0.001***
	**Foliaceae**	**0.01**	**−0.0025**	**0.02**	**0.09**
	Contact	−0.06	−0.16	0.04	0.240
	**Young**	**−0.16**	**−0.33**	**−0.01**	0.04*
	Contact*Young	**0.14**	**0.01**	**0.27**	0.04*
Body size	Intercept	473.61	416.81	529.23	<0.001***
	**Moss**	**4.88**	**2.6**	**7.1**	<0.001***
	Contact	−48.23	−109.61	12.32	0.11
	Young	−49.05	−130.45	26.25	0.21
	Contact*Young	**84.88**	**4.7**	**170.17**	0.04*
*M. brevipes*	Intercept	2.48	1.57	3.24	<0.001***
	**Foliaceae**	**−0.09**	**−0.18**	**−0.01**	0.04*
	Contact	0.29	−0.30	0.93	0.34
	Young	0.72	−0.50	1.87	0.22
	Contact*Young	**−0.81**	**−1.78**	**−0.11**	**0.06**
*D. plantivaga*	Intercept	2.64	2.11	3.18	<0.001***
	**Contact**	**−0.68**	**−1.23**	**−0.04**	0.03*
	Young	−0.14	−0.94	0.54	0.73
	Contact*Young	**0.75**	**−0.07**	**1.58**	**0.07**

A significant positive interaction term indicates an effect of dispersal limitation onto younger tree crowns (a negative term would be consistent with replacement by immigrants after suppression of dispersal limitation). Variable selection by backward elimination (Methods) using P = 0.1 as cutoff for inclusion. The table gives parameter estimates (posterior means, taking into account non-independence in the random effects), Bayesian 95% Highest Posterior Density (HPD) intervals and p values (two tailed, but note that those for “young” and “contact” actually test and confirm one-tailed hypotheses from the Introduction). ‘ns’ indicates no variables met the cutoff criteria. Variables that are at least marginally significant are given in bold. N = 16 crowns * 2 treatments. For simplicity we left out varibles that are not selected into the model and the random factor (means and 95%CL always positive). Note that *M. brevipes* is the smallest among the abundant species, suggesting relatively high dispersal capacity but low performance, and the reverse for the larger *D. plantivaga*.

### Dispersal limitation towards young tree crowns reduced mean body size as exemplified by the two dominant mite species

**Figure 2:**
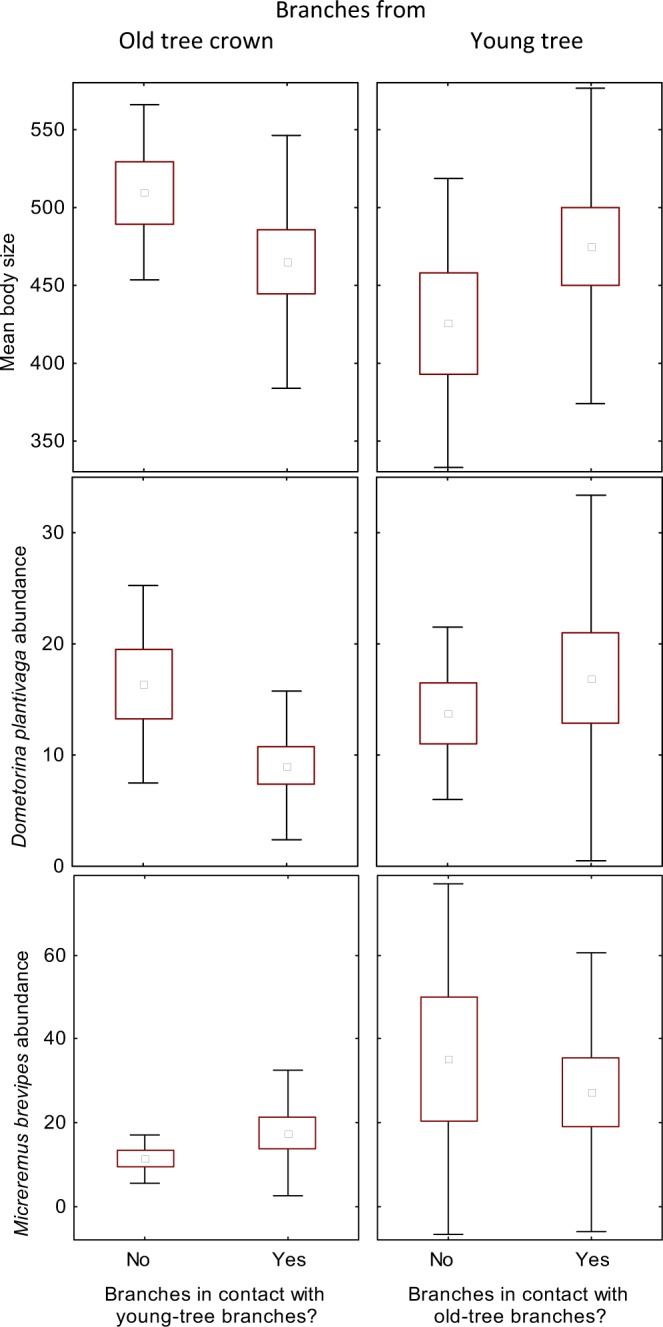
Averages (with standard errors and standard deviations) of body size of oribatid mites, and abundances of the two most common species on experimental branches, *M. brevipes* and *D. plantivaga*, the former being smaller than the latter. For branches not in contact, the average size of mites on branches taken from old trees is strikingly larger than those from young trees. When branches taken from older and younger tree crowns are put into contact, the average body size of mites becomes more similar, at least partly explained by a proportional increase in *D. plantivaga* and a proportional decrease in *M. brevipes* abundance on such young-crown branches put in contact with old-crown branches. Note that the focus is on the interaction ‘contact × age’, which is significant for average body size and marginally significant for abundance of both mite species (see Table 1), rather than on comparisons between individual conditions.

Overall, oribatids on young-crown branches were on average significantly smaller than those on old-crown branches. This relationship significantly reversed when young-crown branches were put in contact with old-crown branches (Table 4, Fig. 2). These shifts could be attributed to shifts in the abundance of the two dominant species: the larger one (*D. plantivaga*, 442.5 µm) tended to be relatively more abundant on young-crown branches that were connected to old-crown branches, while the smaller one (*M. brevipes*, 290 µm) tended to be relatively less abundant on young-crown branches that were connected to old-crown branches (Table 4, Fig. 2).

### Across all species, larger-bodied species were more dispersal limited in colonizing young tree crowns than were smaller-bodied species

**Figure 3:**
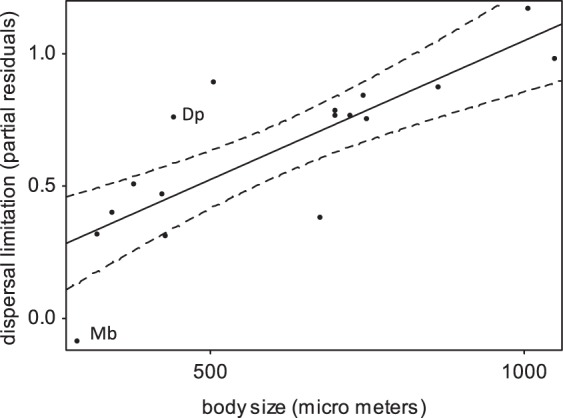
Partial residuals for the effect of body size on dispersal limitation, accounting for the simultaneous effect of the other variables, and the estimated regression line with 95% CI; P = 0.021 (Table 5). Dispersal limitation towards crowns of young trees is higher in species of large body size than in species of small body size. Dispersal limitation was calculated for each species from the interaction between *young-crown-branch* and *contact-with-old-crown-branch* in models including all microhabitat covariates. Dispersal limitation was then related to body size of species, their arboreal life-style and the interaction between the two (Table 5). *M. brevipes* and *D. plantivaga* are marked Mb and Dp, respectively.

A positive interaction [young-crown-branch*contact-with-old-crown-branch] in our statistical model indicates dispersal limitation as abundances increase on branches of the young crown when put in contact with branches of the old crown. Across the entire species pool (representing body sizes of 245–1050 µm) the effect size of this interaction term significantly increased with body size (Table 5, Fig. 3)), demonstrating that larger mite species were more dispersal-limited. Moreover, the effect size also tended to increase with a more arboreal life-style (i.e. more tree- rather than ground-dwelling habits; see Methods) and there was a weaker but still significant interaction between body size and arboreal life-style (Table 5), showing that the body size effect was strongest in arboreal species, i.e. species that would rely fully on aerial dispersal as the ground is not suitable habitat for them. (Note that effect sizes did not relate to species ln-abundance: r_Pearson_ = −0.11, p = 0.65, r_Spearman_ = −0.05, p = 0.84).

**Table 5:** Dispersal limitation is stronger in oribatid mite species that are large. For each species, dispersal limitation is inferred beforehand from a proportionally higher abundance on young- vs old-crown branches where these branches are put in contact (i.e. a positive interaction term [young crown * contact with contrasting age], transformed into an effect size. Methods). The present multiple regression analysis statistically explains dispersal limitation of species by their body size, while accounting for arboreal distribution (i.e. habitat from ground-living to strictly arboreal-living) and its interaction with mean body size. The table gives parameter estimate, beta values (standardized parameter estimates), t and p values (for one-tailed hypothesis). Df = 13, total R² = 0.29. See Fig. 3 for an illustration of the results.

	Parameter	Beta value	T	P value
*Intercept*	−0.55	0.00	−1.91	0.0786
*Arboreal habitat*	0.83	1.54	1.93	0.0380
*Body size*	0.00	1.14	2.26	0.0208
Arboreal habitat * Body size	0.00	−1.66	−1.88	0.0413

## Discussion

By experimentally suppressing dispersal limitation, we provide evidence that, at least during the duration of the experiment, (i) individual tree cowns in a contiguous forest canopy can be habitat islands for bark mites living on peripheral branches, and (ii) dispersal limitation is in part determined by species body size. Specifically, the time available for colonization of a tree crowns appears to influence the size distribution of mite communities on the branches within that crown: Larger mites are less prevalent on peripheral branches of younger crowns than they would be without dispersal limitation (i.e. when young-crown branches are put in contact with old-crown branches). This is true for mean body size, reflecting mainly the smaller and larger among the two dominant species, and also across all species in the regression analysis treating each species as a data point and hence covering a very large range of body sizes. To our knowledge, this is the first study in which dispersal limitation has been manipulated by connecting naturally disconnected patches to study community assembly, instead of cutting and then reconnecting a large habitat patch^24^ or forcing individuals into the isolated patch and hence manipulating recruitment rather than dispersal limitation^20^.

Thanks to our experimental approach, we were able to demonstrate that the effect of tree-crown age on mite community composition is influenced by dispersal limitation (low rate of colonization) while holding local microhabitat parameters constant. The observed effect of crown age does not seem to be due to microhabitat differences between similarly aged branches of young and old trees, even though microhabitat properties are undoubtedly important to bark-dwelling mites^36,39^. Indeed, the microhabitat properties on young tree crowns seem to be highly appropriate for the large-bodied species, as removing dispersal limitation triggered immigration of large bodied species onto branches of young trees, and possibly even displacement of incumbent species of small body-size. Moreover, microhabitats, i.e. cryptogam compositions, were accounted for in all our models, and microhabitat parameters did not differ systematically between branches from young and old crowns. The similar cryptogam compositions also indicate that microclimatic conditions in the trees of origin were similar, as was attempted in our study design. Furthermore, within each of the small experimental replicates, systematic microclimatic variation among branches from crowns from old and young trees is unlikely.

We do not pretend to capture the entire oribatid mite communities or all aspects of assembly of the communities. A tree is much more than the younger, peripheral branches we studied, and some of the remaining parts of a tree may change with its age, such as the bark on the trunk, confounding tree age with microhabitat^26,31^. Younger branches, in contrast, are habitat structures that are less likely to differ between young and old trees, and did not do so in the present study (Tables 2 and 3; but note that microhabitat factors were nevertheless accounted for in our analyses). Moreover, oribatid faunas on such younger branches may be partly distinct from those on the older branches or on trunks where three-dimensional cryptogams are much more abundant and bark crevices are much deeper, resulting in much higher oribatid mite abundances than found in the present study^25,31^. Across time scales longer than our experimentation, exchange of oribatids between younger and older branches might be important for community assembly on the younger branches. For instance, if due to local habitat factors, larger mites were more abundant on the old branches of old than of young tree crowns this might create a pressure on old tree crowns to colonize young branches. So we do not know for sure what ultimately increases abundances of large mites on young branches of old compared to young tree crowns. But our experiment shows that at least temporally this disequilibrium is maintained by limited dispersal between old and young trees.

We also do not pretend that dispersal and performance of poor and good dispersers are the only factors affecting the assembly of oribatid mite communities even on the bark of young branches. In fact, the analyses demonstrate that microhabitats have an effect (Table 4), and other factors such as enemy-free space may exist. Actually, we have no prove that for the specific communities we studied aerial dispersal is a limiting factor in the long run. Albeit multiple studies have demonstrated that many oribatids colonize bark by the air^27,40^, we have not directly studied aerial dispersal as such and whether there are more small than large oribatids floating in the air and “landing” on the branches. The analyses, however, do demonstrate an effect of isolation from old trees and of suppressing this isolation: larger oribatids from older tree-crowns do move onto branches from younger tree-crowns when they are put in contact.

We finally also do not pretend to capture all consequences of dispersal limitation in oribatid mite communities on tree crowns. Future studies could address how mite communities change over time as trees age. This may also include experimental studies of competitive interactions amongst bark mite species of different body sizes and hence different dispersal capacities. Future studies could also address demography to assesss the influence of dispersal on abundances in this system. The present study used mite communities on trees that had established under dispersal limitation over years, but observed consequences of suppression of this dispersal limitation only across several weeks. It is likely that continued dispersal would maintain these short-term consequences. However, other differences among strong and weak dispersers, such as differences in fecundity and type of reproduction, may come into play, and either accelerate or compensate the effects of dispersal alone. Unfortunately, these traits are to our knowledge not known for several of the species we had studied and seemingly do not differ between our two focal species^41,42^. Finally, future studies might assess within-species variation of body size among individual trees^43^. There is intraspecific body-size variation^39^ and local selection might benefit smaller individuals in the dispersal towards younger trees. This selection would hence reinforce the pattern of body-size dependent species-sorting we had observed. Moreover, our study covers a 400% range of body sizes. This can hardly be swamped by intraspecific variation which amounts to only 17 and 23%, respectively, for our two focal species and 26% across all species (results not shown).

We have experimentally demonstrated the existence of dispersal limitation among crowns in large-bodied species, and the patterns we find in community composition allow us to hypothesize, for future testing, by which processes such dispersal limitation affects community assembly on hosts. If dispersal limitation is random, species richness should decline with increasing dispersal limitation because extinctions are only weakly balanced by colonisations. Extinction and colonisation being stochastic, there are no predictable trends in species composition, and total abundance and evenness of abundances remain constant^6,7^. In contrast, under deterministic dispersal limitation and with a dispersal-performance trade-off, species composition would shift from disperser-dominated to performer-dominated communities, while species richness and total abundance are expected to remain unchanged. Communities on hosts of an intermediate age should have highest evenness, with a mix of dispersers and competitors. In contrast, if competition is unimportant, performers will not replace dispersers. Species richness and total abundance will increase from communities on young to such on old hosts due to the arrival of poor dispersers The observed high relative abundances of large species with poor dispersal capacity on old tree crowns, without a decrease in overall species richness or in overall abundance, is hence consistent with trait-driven, i.e. deterministic, dispersal assembly in which good performers tend to competitively replace good dispersers. The relatively higher evenness observed on branches from crowns of older trees (without contact to young-crown branches) might then reflect the stage in which both superior dispersers and superior performers are still present and have more balanced abundances than on either younger or even older tree crowns (at least during the duration of the experiment).

The results suggest a decline of smaller bodied species after successful immigration of large bodied species. This indicates that large bodied species perform well on crowns of young trees, and perhaps partly outcompete small-bodied species. The obvious question is how competition might operate between organisms occurring at such relatively low densities. We do not have a definite response. It should however be kept in mind that the surface truly available for a microarthropod on an exposed structure like a branch in a tree crown can be much smaller than the total surface, being restricted to microclimatic shelters and their direct vicinity^44^. Moreover, often only specific parts of the lichens are useable, such as only a single layer of phycobiont cells in the wetted lichens^33^. With such restrictions of resources, it may not be surprising to find patterns consistent with partial competitive replacement of small species.

We show that, at least during the duration of the experiment, dispersal limitation does not require large spatial distances. Habitat islands such as tree crowns that are spatially adjacent from the point of view of humans can pose severe dispersal problems from the point of view of many organisms. In addition, we show that patch age is not only important at the geological time-scale of oceanic islands or entire forest patches across centuries, but also at the scale of individual forest tree-crowns across few decades. Given that there are other flightless biota in tree crowns besides oribatid mites (e.g. flightless moths, scale insects), dispersal limitation might be of general importance in determining arthropod communities in tree-crowns within a contiguous forest canopy. These results add to the growing body of evidence that principles of island biology can be applied to individual trees within a contiguous canopy, where island size, age or phylogenetic isolation affect community structure as well as phenotypes of arthropods^43,45–47^. More generally, results from our model system suggest that, first, the dispersal limitation of large mites towards young trees might create a refuge for cryptogams from the larger mites and their more destructive mouth parts^33^. Second, body-size mediated trade-offs between dispersal and performance are important in deterministic community assembly on habitat islands. Finally, it appears possible that, opposite to common wisdom on macroscopic animals^12–14^, evolution of large body sizes in flightless plant-dwelling invertebrates^5^ might be constrained by a dispersal handicap: large body size makes these invertebrates fall too fast to colonize new hosts through aerial dispersal.

## Methods

### Sampling design and field protocol

The experimental approach of this study was to sample branches from younger and older tree crowns and then put the two in contact, in order to suppress the dispersal limitation onto the branches of young crowns. The communities that establish without dispersal limitation are then compared to those establishing without contact, i.e. with dispersal limitation (rather than quantifying numbers of immigrants on virgin substrates)^40^. Specifically, if certain mite species are more abundant on young-crown branches put in contact with old-crown branches, this indicates existence of dispersal limitation from old to young crowns. If dispersal had not been limited, these mites would have moved to the branches of the younger crowns before they were put in contact. In contrast, any general differences in mite communities between branches from young and old crowns, found both on the branches that had been put in contact and those that had not, are independent of dispersal limitation and hence attributable to differences in niche conditions among young- and old-crown branches. We strived to avoid differences in niche conditions on the experimental branches. The age of the experimental branches *per se* did not differ between tree crowns, and in each pair, trees were of similar height and shape and directly adjacent to each other. Finally, the analyses accounted for key microhabitat parameters.

Specifically, we selected 8 pairs of young and old *Quercus petraea* trees in the Forêt de Rennes (48°11′N, 1°34′W, described in^45^), France, where a young tree is defined as one with a trunk circumference at breast height of 40–50 cm (corresponding to approximately 30 years) and an old tree is one with 100–120 cm (>60 years). Tree crowns from the same pair were of similar height and growing in the same, oak-dominated, forest parcel, of approximately 17–20 m canopy height, but were not in direct contact with each other. For technical reasons (ease of putting branches in direct contact), tree crowns had to have straight branches available at a height of approximately 8 m to be selected for the experiment.

For each of these 8 pairs of trees, we established the following experimental design. Each experimental setup consisted of 6 branches (standardized as explained below): one branch from the young crown not in contact with a branch from the old crown, one branch from the old crown not in contact with a branch from the young crown, and two pairs of old-crown + young-crown branches put into direct contact to suppress dispersal limitation onto the young-crown branch (Fig. 1). We decided to have two of these “young-old in contact” treatments as connecting branches might induce some degree of perturbation of microenvironments and their oribatid mite faunas and thus result in a higher variance of these faunas. Putting into contact *per se* is a treatment where branches might, for instance, mutually shade each other. Such a possible mutual influence of young-crown and old-crown branch on each other would be not depend on branch age, it should hence not affect what we tested for: a change of community structure or body-size depending on branch age. Moreover, analyses accounted for any effect of putting branches into contact by using ‘contact’ as a covariable (see below).

Experimental branches were 60 cm long, had diameters between 2.2 and 3.2 cm and represented close-to terminal sections of larger branches (we avoided the most terminal sections as these had less cryptogams). When handling the branches, we avoided any contact of the branches with the litter to prevent colonization by litter oribatid mites. Branches were connected by tape, ensuring that branch faces that were initially exposed to sun (i.e. lichen-covered faces) remained exposed, whereas faces that were initially shaded (covered by algae only) remained shaded, i.e. oriented towards the other branch to which it was put in contact. The two branches were connected by floral foam. Laboratory pilot observations had shown that bark mites move freely on floral foam and that the distance between two branches can be easily traversed by the mites within a day. Indeed, oribatid mites can move distances of decimeters within hours^48,49^. The different branches (two controls, two pairs of young-old-contact treatments) were firmly fixed on a wire net at each end so that the position of the branches could be controlled (Fig. 1). Points of attachment were covered with double-sided adhesive tape to prevent any emigration of mites on the wire. For each of the pairs of trees the setup was then installed in a (non-sampled) mature sessile oak (*Quercus petrea*) crown at a height of 8 m (the same height branches were collected from) and fixed with ropes from above as well as below, ensuring that the setup did not touch other branches from the canopy. The experiments were installed between 14 and 23 March 2007 and were terminated between 3 and 6 April.

### Extraction of oribatid mites and characterization of mite species and branch habitats

Branches were washed with water at high pressure for several minutes, ensuring that all epiphytes including foliose lichens and mosses were flushed off the bark. The water was then filtered, and the filter was stored in 70% alcohol and (after storage in a freezer) washed into a small vessel. The oribatid mites were then separated from the organic debris using flotation in heptane as described by Walter *et al*.^50^ and Kethley^51^. This method does not seem to select against small species (correlation between abundance and body size r = −0.34, p = 0.18), even though it might be less efficient for smaller soil-dwelling mites, but there was no soil on the harvested branches. Specimens of all stages were identified by S. Woas following Weigmann^39^, and specimens were deposited in the Staatliches Museum für Naturkunde Karlsruhe. Most nymphs could be identified to species level. Numbers of individuals per branch could be treated as ‘abundance’ because branch size was standardized: all had a cylinder shape and were covered mainly by crustose cryptogams, without any three-dimensional cryptogams such as fruticose lichens. We found densities of on average 49 individuals on a 60 cm branch, which appears to be not unusual^33,52^, and even high compared to other studies focussing on an almost entirely crustose cryptogam cover^38^.

We characterized mite species by their adult body sizes as the mean between extremes given in Weigmann^39^. Weigmann^39^ (and others) only provide body length, but length is a good proxy for size given that shapes are relatively similar and lengths very different among species. Our approach ignores intraspecific variation, which however is limited in oribatid mites^39^ and distinctly lower than the 50% variation in body length between the two focal species (*D. plantivaga* and *M. brevipes*) and the 400% variation among all species. We also characterized mites by their habitat preferences into degrees of arboreality following Weigmann^39^: species were ranked as 0 = mainly ground living; 0.5 = both living at the ground (notably dead wood) and on bark/cryptogams; 0.75 = living in cryptogams or mainly arboreal; and 1 = arboreal. We note that species using the “arboreal” habitat are all cryptogam feeders^33^; our two focal species are hence both cryptogam feeders. We explored in the analyses how modifications of this ranking might affect the results and found that it doesn’t, see “Statistical analyses” section below. A list of species with their average abundances and their body size and degree of arboreality is given in Table 1.

Microhabitat properties on the branches were characterized by quantifying the percentage coverage of the tree bark by different cryptogam types (algae, crustose lichens, foliose lichens, mosses, bare bark, similar to^53^). The type of such cryptogams is essential for many oribatid mite species as absence of the “right” microhabitat cannot be compensated by a high diversity of the remaining microhabitats^32,53^. We stress that we accounted for microhabitats as covariables when testing the predictions of our hypotheses (see below) and we provide average coverages of different microhabitats on different branches in Table 2. We also stress that we strived to minimize such differences among experimental branches and indeed compositions of microhabitats did not differ significantly between young and old-crown branches (Tables 2 and 3).

### Characterization of dependent variables

For each branch, we determined the abundance of every mite species. Average body size per branch was calculated for each sample as the mean across the sizes of species weighted by species abundance. Such abundance-weighted community-wide averages have proven a useful measure of trait filtering and avoid bias due to species only represented by very few individuals^54^. These averages strongly depended on the two most abundant species *Micreremus brevipes* and *Dometorina plantivaga* (46 and 27.8%, respectively, of the total 2363 individuals), which represent distinct mean body sizes (290 vs 442.5 µm). In accordance with the decrease in aerial dispersal ability with increasing body size of mites (Introduction)^40^, found the smaller *M. brevipes* to float in large numbers in the air while the larger *D. plantivaga* is almost absent in aerial plankton samples. We calculated total mite abundance and species richness for each experimental unit. We calculated the evenness of species abundances on a given branch using Pielou’s parameter^55^.

### Statistical analyses

Community analyses were performed using Bayesian mixed-effect generalized linear models run in R^56^ using the package MCMCglmm^57^. Mixed-effects analysis accounts for the lack of independence (and hence reduced degrees of freedom) between branches from the same tree crowns, as well as pairs of branches in contact, which would be expected to be more similar than random if some exchange of individuals had occurred. A weak prior was employed, with the default prior variance (V) = 1, and ‘degree of belief’ parameter (nu) = 0.002 for all fixed and random effects. Models were run for 10k iterations after a burn-in of 3k, and with a thinning interval of 10 as standard. For several traits with relatively low sampling efficiency for certain variables, iterations were increased to 90k after a burn-in of 10k.

We tested the response variables average body size, total abundance, species richness, species evenness, *D. plantivaga* abundance, and *M. brevipes* abundance using multiple regression with variable selection. The full model included the additive effects of the 5 microhabitat variables (percent cover of different types of cryptogams) plus the additive and interaction effects of age of the crown-of-origin (young vs. old) and the treatment (contact with branch from crown of contrasting age vs non-contact) as fixed effects. The values of response variables may be correlated by crown-of-origin and through physical contact with the other branch for paired branches. Hence, ‘crown’ and ‘pair’ were included as random effects. Backward elimination was then employed on the fixed effects to identify the best model, using p < 0.1 as the cutoff for inclusion. (We note that best subset selection led to almost identical results for the variables of interest, i.e. age, contact, age * contact.). Random effects were retained in all models. Poisson errors were used for species richness and for abundance of each of the two dominant species, and Gaussian errors were used for the other dependent variables. Note that MCMCglmm automatically estimates the residual variances for Poisson models, so no additional assumptions need to be made about mean-variance scaling.

Finally, we performed an analysis to test whether there is a general relationship between the body size of mite species (ranging from 245 to 1050 µm), and their dispersal limitation towards crowns of young trees. First, we conducted a separate ordinary least squares regression analysis explaining abundance for each of the species and identified the effect size of the interaction term young-crown branch * contact with old-crown branch. A positive interaction term is evidence for dispersal limitation onto young crowns as abundances increase once the branch from the young crown is put in contact with that from the old crown. A negative interaction term, in contrast, indicates decline on a young-crown branch after contact with old-crown branch, possibly due to replacement by immigrants from old crown branches. We included all five microhabitat variables as covariates in the analyses. We did not perform any variable selection as the goal was to use the same model for all species, resulting in *young-crown* * *contact with-old-crown* interaction terms that are comparable among species. We retained the t values of these interaction terms and transformed them into the effect size r as r = sqrt (t²/(t² + (df − 4)))^58^. We then performed a cross-species meta-analysis to test the one-tailed hypothesis that dispersal limitation (effect sizes of *young-crown* * *contact-with-old-crown*) increases with the body size of the species. Significance is hence tested across species and not within each of the species, which would not be possible for the rarer species^59^. To better identify the signal of body size, we also included *arboreal habitat* (species’ ranking of degree of arboreality, see above) and the interaction *body size* * *arboreal habitat* into the model as species that are also ground-living might colonize a young crown from the ground, and thus not experience individual trees as habitat islands. Residual plots indicated one outlier species introducing strong variance heterogeneity, *Ramusella elliptica*, which was hence excluded from the analysis (this species was present on only a single tree). Habitat ranking was ordinal and may therefore be somewhat imprecise. We hence explored whether changing the precise values of ranks changes the result on body size: (i) we log transformed and square-transformed the habitat rankings, with no change in the results on body size (p remained < 0.05). (ii) We recreated 50 habitat rankings introducing in each a minor error of <= +/− 0.125 (i.e. ¼ of the total range of the values). The resulting analysis yielded the expected, one-tailed effect of body size at p ≤ 0.05 in 49 out of 50 cases. We note that an alternative approach to this analysis was to weight the species by their abundance, leading to qualitatively the same, significant effect of body size, and without any particular species showing extreme residuals.

### Data availability

The data will be made available in the Dryad online data repository (https://www.datadryad.org).
